# Rapid and Automated Method for Detecting and Quantifying Adulterations in High-Quality Honey Using Vis-NIRs in Combination with Machine Learning

**DOI:** 10.3390/foods12132491

**Published:** 2023-06-26

**Authors:** José Luis P. Calle, Irene Punta-Sánchez, Ana Velasco González-de-Peredo, Ana Ruiz-Rodríguez, Marta Ferreiro-González, Miguel Palma

**Affiliations:** Department of Analytical Chemistry, Faculty of Sciences, University of Cadiz, Agrifood Campus of International Excellence (ceiA3), IVAGRO, 11510 Puerto Real, Spain; joseluis.perezcalle@uca.es (J.L.P.C.); irene.punta@uca.es (I.P.-S.); ana.velascogope@uca.es (A.V.G.-d.-P.); ana.ruiz@uca.es (A.R.-R.); miguel.palma@uca.es (M.P.)

**Keywords:** honey, adulteration, machine learning, visible near infrared spectroscopy, support vector machine, random forest

## Abstract

Honey is one of the most adulterated foods, usually through the addition of sweeteners or low-cost honeys. This study presents a method based on visible near infrared spectroscopy (Vis-NIRs), in combination with machine learning (ML) algorithms, for the correct identification and quantification of adulterants in honey. Honey samples from two botanical origins (orange blossom and sunflower) were evaluated and adulterated with low-cost honey in different percentages (5%, 10%, 15%, 20%, 25%, 30%, 35%, 40%, 45%, and 50%). The results of the exploratory analysis showed a tendency to group the samples according to botanical origin, as well as the presence of adulteration. A supervised analysis was performed to detect the presence of adulterations. The best performance with 100% accuracy was achieved by support vector machines (SVM) and random forests (RF). A regression study was also carried out to quantify the percentage of adulteration. The best result was obtained by support vector regression (SVR) with a coefficient of determination (R^2^) of 0.991 and a root mean squared error (RMSE) of 1.894. These results demonstrate the potential of combining ML with spectroscopic data as a method for the automated quality control of honey.

## 1. Introduction

Honey is a sweet and natural substance produced by bees from the nectar of flowers. Nowadays, this product is very popular and appreciated by consumers due to its nutritional benefits and its antioxidant and antimicrobial properties [1]. However, as a result of its popularity and high value, it is one of the products most prone to fraud. In fact, it has been reported by the European Commission as one of the ten most adulterated foods in Europe [2]. For this reason, and in order to ensure the quality and safety of honey, most countries have established different regulations and laws. These regulations specify the requirements that honey producers and their products must meet, as well as the conditions for labeling, packaging, and marketing. According to directive 2014/63/EU of the European parliament, honey is a pure product where the addition or substitution of any substance is prohibited [3].

The most frequent adulterations include the addition of other cheaper and low-quality sweeteners such as high fructose corn syrup, corn sugar syrup, inverted sugar syrup, and cane sugar syrup [4]. However, adulterations by blending an expensive honey with a low-cost honey are becoming more and more frequent. To exemplify, both Burzyan and Manuka honeys are known for their high price, so they would be prone to adulteration [5]. Nevertheless, unifloral honeys, in general, are the most adulterated, because these are produced from a defined botanical source, which confers distinctive organoleptic properties, and are more valuable than multifloral honeys [6].

Therefore, it is crucial to have methodologies to detect honey adulteration. The most widely used methods for this purpose include gas chromatography (GC) and liquid chromatography (LC) [7]. LC has reported excellent results in guaranteeing the authenticity of honey [8,9], as well as in the detection of different adulterations such as starch syrup [10], rice syrup [11], and fructose and saccharose syrups [12], among others. GC has also been successfully applied for similar purposes [13,14,15]. However, both GC and LC analyses require long sample preparation times and specific method optimization. Additionally, the chromatographic methods need several chemicals and usually produce waste. Therefore, other methodologies have been used in recent years to detect various types of adulteration in honey, including ion mobility spectrometry (IMS) [16,17,18], nuclear magnetic resonance (NMR) [19,20], DNA-based techniques [21,22], and pollen visualization [23,24], among others. Among them, pollen visualization stands out, as it is a fast and cheap methodology that can be combined with machine learning algorithms, allowing for the characterization of honeys in an objective way [23].

Spectroscopic techniques are also a good alternative since they have many advantages such as speed, reliability, lower cost, no reagents, and no waste. Most studies focus on the use of attenuated total reflectance–Fourier transform infrared spectroscopy (ATR-FTIR), which has reported excellent results for the characterization of honeys according to their botanical origin [25] as well as the detection of a multitude of adulterants [26,27,28,29]. The use of near infrared spectroscopy (NIRs) has also been widely employed to identify the botanical origin of honey [30,31,32], as well as to detect different adulterations by the addition of sweeteners [33,34,35,36,37,38,39,40]. However, to the author’s knowledge, it has never been employed to quantify the percentage of adulteration in honeys resulting from blends with lower quality and lower-price honey.

NIR spectra generate a huge amount of data, and the information is not easily interpretable. Therefore, NIR spectra are often combined with machine learning (ML) models. Additionally, in complex samples such as honey, there is a significant overlapping of the bands, so strategies for preprocessing the spectra are commonly necessary. In this sense, various mathematical corrections have been previously applied to NIR spectra, including orthogonal signal correction, the first derivative, or smoothing filters such as Savitzky–Golay [41]. These corrections avoid the band overlapping and reduce the signal-to-noise contributions, thus improving the performance of the ML models. Another very important consideration is the selection of the working spectral range, as it simplifies the ML models by making them more interpretable and reducing the computational cost of training. There are different methods for feature selection. Relatively recently, genetic algorithms or the Boruta algorithm coupled to the NIR spectra have been used [35,42].

On the other hand, most studies combine NIR spectra with parametric ML models. Specifically, these use linear discriminant analysis (LDA) for classification and partial least squares regression (PLS) for quantification. However, the use of other nonparametric techniques such as support vector machines (SVM) and artificial neural network (ANN), as well as random forest (RF) models are less commonly used, even though they usually offer better results [32,33,35] in complex matrices.

Therefore, this study aims to develop a method based on Vis-NIR spectroscopy in combination with ML models for the automated detection and quantification of adulterations of unifloral honeys from different botanical origins (orange blossom and sunflower) blended with low-cost honeys. Both the most common parametric models (PLS and LDA) and nonparametric models based on SVM and RF were evaluated.

## 2. Materials and Methods

### 2.1. Samples

As unifloral honeys are often adulterated with multifloral honeys or with cheaper unifloral honeys [6], samples of high-quality honey from two different botanical origins (orange blossom and sunflower) were used for this study as monofloral honeys to be adulterated. In order to increase the heterogeneity of the sample set, the unifloral honey samples were obtained from different geographical origins (South Spain, specifically from Huelva, Cordoba, Sevilla, and Cadiz—2019 campaign). It should be noted that these samples belonged to protected designation of origin (PDO) and were provided by Spanish regulatory entities to guarantee their authenticity and quality. A total of 8 orange blossom samples and 3 sunflower samples were obtained. The adulterant was prepared by blending 4 low-cost commercial honeys in equal proportion, as follows: 2 of them from multifloral (1 of Spanish origin and 1 of non-European Union origin), 1 from lavender (non-European Union origin), and 1 from rosemary (Spain origin). These honey samples were purchased in different markets. This choice was made in this way to increase heterogeneity (i.e., to cover a wider range of possible adulterations) and because their lower price compared to orange blossom and sunflower honeys makes these low-cost honeys more prone to be used to adulterate high-quality honey.

The samples were labeled with an alphanumeric code where the number refers to the sample identifier, and the letter indicates the botanical origin (“OB” for orange blossom and “S” for sunflower). In addition, they were analyzed in duplicate; therefore, the fifth orange blossom sample would be labeled as 5OB_R1 for the first replicate and 5OB_R2 for the second. The total number of unadulterated samples analyzed was 22 (16 orange blossom honey samples and 6 sunflower honey samples).

### 2.2. Adulteration

Adulterated samples were prepared independently for the two types of honey using the orange blossom mixture and the sunflower mixture. From these blends, adulterated samples were prepared by adding the adulterant sample (mixture of the low-price honeys) in the following proportions: 5, 10, 15, 20, 25, 30, 35, 40, 45, and 50%. Again, the samples were analyzed in duplicate, making a total of 40 samples (2 botanical origins × 10 adulteration percentages × 2 replicates). A total of 8 additional samples (4 in duplicate) corresponding to the mixture of orange blossom and sunflower honeys, used as 0%, were also analyzed. Therefore, the total number of samples in the adulteration process was 48. Samples were labeled with the botanical origin (“OB” or “S”), followed by the percentage of adulteration. Thus, a 5% adulterated sunflower sample would be labeled as S_5_R1 for the first replicate and S_5_R2 for the second replicate.

### 2.3. Visible near Infrared Spectroscopy (Vis-NIRs)

For the analysis of the samples, the FOSS XDS Rapid Content™ spectrometer (FOSS Analytical, Hilleroed, Denmark) was used. This equipment has a unique light beam analyzer, and spectra were acquired from 400 to 2500 nm with a resolution of 0.5 nm. The main objective of this study was to develop a method for the characterization of honey samples based on a nontarget analysis, i.e., without the identification of individual compounds. For this reason, the whole spectra, which include the signals obtained at 4200 measured wavenumbers, were used as an overall profile of the sample and 10 g of the sample was analyzed in 10 mL vials. For each sample, two spectra were recorded, and the result was the average of both. The total analysis time per sample was 30 s.

### 2.4. Data Analysis

ML classification models used for the detection of adulteration included: LDA, SVM, and RF. Similarly, the ML regression models used for the quantification of adulterant were PLS, SVR, RF, and shrinkage methods (lasso, ridge, and elastic net). All data analysis was performed using RStudio software v.4.2.2 (RStudio Team 2022, Boston, MA, USA). The data were stored in D_nxp_ matrices where *n* denotes the number of samples and *p* the number of predictors. The main RStudio packages used were ggplot2 [43] for graphical representations; prospectr [44] for data preprocessing consisting of applying the first derivative and Savitzky–Golay filter; factoextra [45] for hierarchical cluster analysis (HCA); caret [46] for creating different machine learning models for both classification and regression; and Shiny [47] for application development.

## 3. Results

### 3.1. Exploratory Analysis

**Figure 1:**
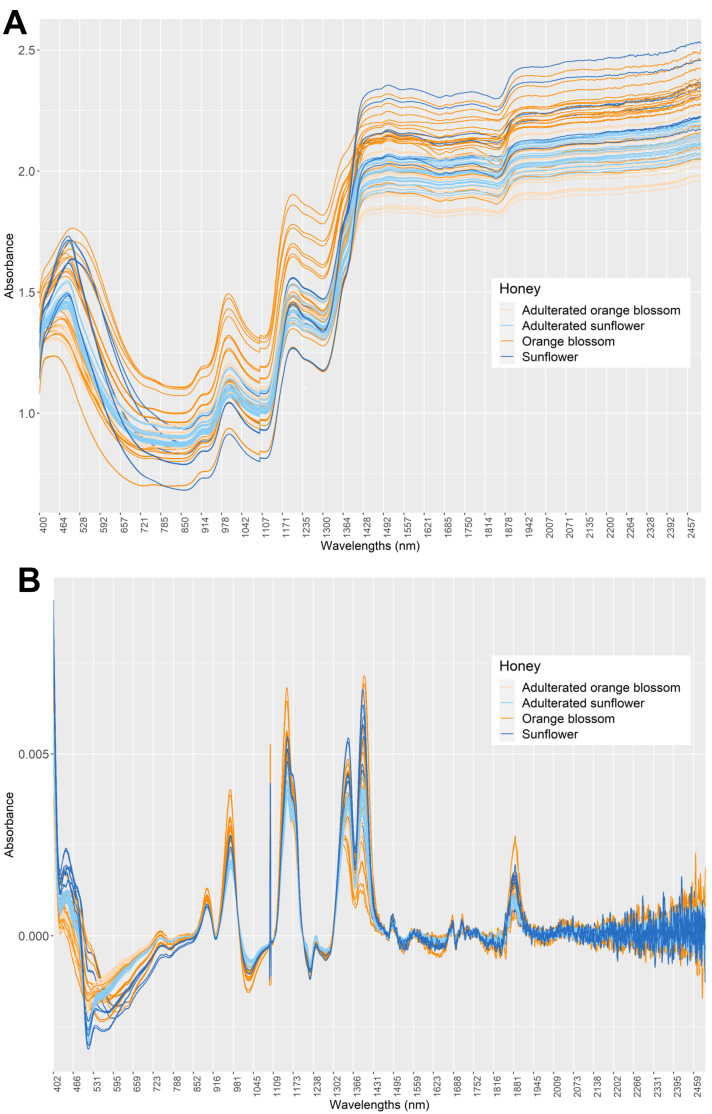
(**A**) Representation of the raw spectra obtained for all honey samples (D_70×4200_) analyzed by *Vis*-NIRs. (**B**) Resulting spectra after applying the first derivative and Savitzky–Golay filter. All samples have been color-coded according to their botanical origin and adulteration status: adulterated orange blossom samples colored in light orange, nonadulterated orange blossom samples colored in dark orange, adulterated sunflower samples colored in light blue, and nonadulterated sunflower samples colored in dark blue.

First, it must be noted that both the honey samples and the adulterants contain the same major compounds, i.e., sugars and water; therefore, high similarity among all Vis-NIR spectra can be expected. In this first stage, the objective was to determine if there was a tendency for samples to be grouped according to their botanical origin (orange blossom and sunflower) as well as the level of adulteration by using the spectroscopic signals from minor compounds in the different samples. For this reason, the complete data matrix (D_70×4200_) consisting of the 70 samples (nonadulterated and adulterated) and the 4200 variables (wavenumbers) was used. In Figure 1A, the raw spectrum of all samples was represented based on their botanical origin and the presence/absence of adulteration: adulterated orange blossom samples (light orange), nonadulterated orange blossom samples (dark orange), adulterated sunflower samples (light blue), and nonadulterated sunflower samples (dark blue).

As can be seen in Figure 1A, the bands are wide and there is a significant baseline shift in all the samples. This phenomenon can lead to confusion since spectra of the same type of honey sample show different intensities; however, these differences are due to the shift of the baseline. As it is not desirable to find patterns on samples suffering from these phenomena, it becomes necessary to apply a data pretreatment strategy. In this case, the first derivative and a Savitzky–Golay smoothing filter (3rd degree polynomial and window size of 11) were calculated. In this way, the first derivative helped to identify changes in the spectral slope, enabling the detection of subtle variations and distinguishing overlapping peaks. On the other hand, Savitzky–Golay smoothing filters were employed to reduce noise and improve the signal-to-noise ratio in spectroscopy data while preserving the overall shape of the spectral peaks. Overall, the joint use of the first derivative and Savitzky–Golay smoothing filters in spectroscopy data analysis offers a powerful approach to enhance data interpretation, enabling the detection of subtle changes and providing a better visualization of spectral features. Figure 1B shows the resulting spectra after applying this pretreatment. As can be seen, several wavelengths at the end were eliminated, reducing the data matrix from 4200 to 4190 variables (ranging from 402.5 nm to 2491.5 nm). The baseline has also been reduced. However, visually finding generalizable patterns according to the type of honey is a very difficult and subjective task. In certain regions of the spectrum, there appears to be differences between some groups. For example, in the region from 1100 to 1200 nm, nonadulterated orange blossom samples have higher intensities than the adulterated sunflower and orange blossom samples. However, the complexity of the matrix is too high, so it is impossible to distinguish the samples by visual inspection. For this reason, it is necessary to use ML algorithms to automate and facilitate the understanding of the data structure.

In this case, an unsupervised algorithm was used to evaluate the clustering tendency of the samples, known as HCA. To carry out this analysis, the complete data matrix (D_70×4190_), corresponding to all samples after applying the first derivative and Savitzky–Golay smoothing filter, was used. In addition, the Manhattan distance and Ward’s method were chosen. The selection of the method was based on the correlation between the cophenetic distance of the dendrogram (height of the nodes) and the original distance matrix. Different methods (single, complete, average, Ward’s, and centroid) were evaluated, and the values are shown in Table S1 (Supplementary Materials).

**Figure 2:**
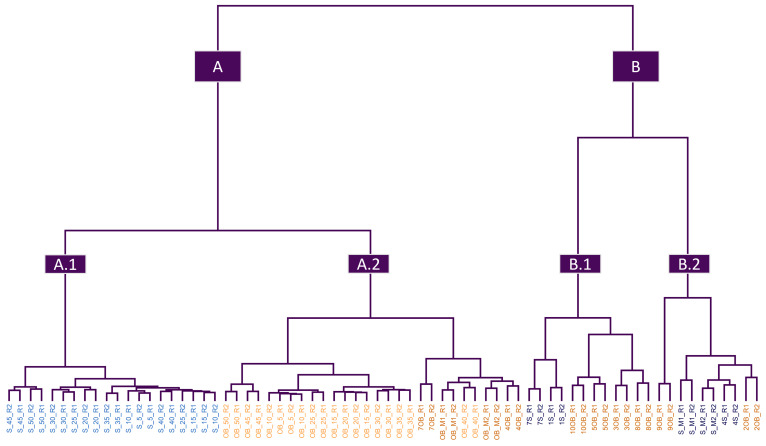
Dendrogram resulting from the HCA analysis, using Manhattan distance and Ward’s method. All samples and spectra resulting from applying the first derivative and the Savitzky–Golay filter (D_70×4190_) have been used. The name of the samples has been recolored based on their botanical origin and adulteration. The samples are labeled with an “OB” for orange blossom (colored dark orange for nonadulterated and light orange for adulterated) and “S” for sunflower (colored dark blue for nonadulterated and light blue for adulterated).

Although all the methods provided similar results, the Ward’s method proved to be the most appropriate with a correlation value of 0.9053. Thus, the dendrogram shown in Figure 2 accurately reflects the true similarity between the observations. In addition, to facilitate the interpretation of the dendrogram, the two main groups have been highlighted, and the samples have been colored based on their botanical origin and the presence/absence of adulteration.

As can be seen, the dendrogram has been divided into two main clusters called “A” and “B”. Cluster A is divided into two subgroups: Cluster A.1, which exclusively contains all adulterated sunflower samples (colored light blue), and Cluster A.2, which contains all adulterated orange blossom samples (colored light orange), but also some nonadulterated orange blossom samples (colored in dark orange). These results show a strong tendency for grouping based on whether the adulterated sample was from sunflower or orange blossom. On the other hand, Cluster B is divided into two subgroups (“B.1” and “B.2”), which contain the remaining unadulterated orange blossom samples and all the unadulterated sunflower samples (colored pink). Focusing on Cluster B.1, it can be observed that it is divided again into two groups, distinguishing sunflower samples from orange blossom ones. In the case of Cluster B.2, the distinction is not so clear, and some sunflower samples appear mixed with orange blossom samples.

These HCA results demonstrate that there was an influence on the Vis-NIR spectra of the samples based on the presence of adulteration, as well as on the botanical origin. However, this grouping tendency was not perfect, as some unadulterated orange blossom samples were included with the adulterated ones. For this reason, and with the aim of developing mathematical models that allow for the prediction of future samples, it is necessary to use supervised ML algorithms.

### 3.2. Classification Models for Adulterant Detection

In supervised analysis, algorithms are trained with a set of previously labeled samples. In this case, the objective was to determine if a honey sample was adulterated or not. Therefore, two groups were established a priori: “Non-Adulterated” where all nonadulterated samples of both sunflower and orange blossom honey were included, and “Adulterated” in which adulterated samples of both sunflower and orange blossom honey at different percentages were included.

The ML models tested included LDA, SVM-lineal, RF, and SVM with radial basis function (SVM-RBF). Prior to training the models, the complete dataset (D_70×190_) was divided into two subsets. The first one was the training set, which consisted of 75% of the samples and was used for model creation. The second subset was the test set, which consisted of the remaining 25% of the samples and was used to validate the model. It should be noted that this subset did not participate in the creation of the models at any point; therefore, the error obtained will be unbiased and quite close to what would be obtained in a real situation. Importantly, the division was executed in a well-balanced manner, guaranteeing that the validation set consisted of samples from the two types of honeys and different percentages of adulteration, in the same proportion as in the training set. Furthermore, the hyperparameter optimization of the algorithms has been performed by 5-fold cross-validation on the training set itself, in order to avoid overfitting. This validation involves dividing the data into five equal-sized folds. Therefore, the model is trained and evaluated five times, with each iteration using a different fold as the validation set while the remaining four folds are used for training. This process allows for a comprehensive evaluation of the model’s performance by ensuring that all data points are used for both training and validation. The final evaluation is based on the average results obtained from the five iterations, providing a robust estimate of the model’s performance and its ability to generalize to unseen data. Furthermore, this validation is used to ensure that the hyperparameter optimization does not overfit the models, leaving the test set to check the real error. Table 1 summarizes the accuracy obtained for the different models.

#### 3.2.1. Support Vector Machines (SVM) with Radial Basis Function (RBF)

The Gaussian kernel function was selected for the SVM-RBF algorithm, requiring the prior optimization of two hyperparameters: the cost (*C*), which controls the penalty for misclassified observations, and gamma (*γ*), which regulates the behavior of the Gaussian kernel. Both parameters were optimized using a grid search method with exponential growth, as previously described [48]. Therefore, to find the best combination of hyperparameters, different values within a specific range (from −10 to 10 in increments of 0.5) were tested for log_2_*C* and log_2_*γ*. Therefore, a total of 8405 models were trained, resulting from the combination of 41 different values for *C* and 41 different values for *γ*, each evaluated using 5-fold cross-validation. The combination that achieved the highest average accuracy in the 5-fold cross-validation was considered the optimal choice. The best results were obtained for log_2_*γ* = −9.5 (*γ* ≈ 1.38·10^−3^) and log_2_*C* = 2 (*C* = 1). It is worth noting that the low value of *γ* suggested that the separation boundaries were practically linear. With this combination of hyperparameters, an accuracy of 100% was achieved both in the training and test sets.

#### 3.2.2. Lineal Support Vector Machines (SVM-Lineal)

As mentioned earlier, the low value of *γ* suggested a linear separation, and for this reason, a linear SVM model was fitted. In this case, the only hyperparameter to be adjusted was the cost (*C*), for which values were tested again in the range from −10 to 10 in increments of 0.5 for log_2_*C*. Thus, the total number of models trained was 205 (41 value of C × 5 folds). The best accuracy results for the 5-fold cross-validation were obtained for a value of log_2_*C* = 6.5 (*C* ≈ 9.77·× 10^−4^). The accuracy with this value was 100% both in the training and test sets.

#### 3.2.3. Lineal Discriminant Analysis (LDA)

In the LDA model, there is no hyperparameter that needs to be adjusted beforehand. Therefore, the model was created directly with the training set and achieved an accuracy of 100% for it and 94.12% for the test set. In this case, a 5% adulterated orange blossom sample was classified as nonadulterated, which must be due to the low level of adulteration. However, although the performance of this model was quite good, it was inferior to the results obtained by the SVM.

#### 3.2.4. Random Forest (RF)

In the RF model, there are mainly two hyperparameters that must be adjusted. These hyperparameters are the number of trees (*ntree*) and the number of variables randomly evaluated before each partition of an individual tree (*mtry*). For classification problems, it is recommended to use a value of *mtry* equal to the square root of the total number of predictors; therefore, its value was 64 (4190 variables) [49]. The value of *ntree* was kept at 500, as it was a large enough number for the error to stabilize. With this combination, a 100% classification was achieved again both in the training and test sets.

In summary, the best performing models in our framework were RF and SVM. Neither of them achieved any error in either the training or the test set. Previous studies in adulteration analysis with data generated using NIRs have reported better results when using SVM models, rather than LDA [50], while others report similar results [30]. In addition, a previous study on the detection of the adulteration of honeys with sweeteners using Vis-NIRs reported excellent results using LDA [36]. In this case, any of the four models could be applicable for detecting adulterants in orange blossom and sunflower honeys. Following the principle of parsimony, the SVM with a Gaussian kernel would be discarded due to its greater complexity, as it did not offer better results than the linear. However, it could also be used since the complexity of the model was not a drawback for the purpose of this research. Furthermore, aside from the aforementioned algorithms, other authors such as Arash Heidari et al. have recently used other deep learning algorithms for various objectives, yielding exceptional outcomes [51,52]. Although the performance was excellent, it is important to highlight that analyzing a larger set of samples would contribute to enhancing the robustness of the models. This approach would encompass greater variability and mitigate the impacts associated with sample preparation variations, as well as those associated with the Vis-NIRs analysis itself.

### 3.3. Regression Models for Adulterant Quantification

Once the models capable of detecting whether a honey sample was adulterated or not were obtained, the next step was to determine the percentage of adulteration. To achieve this, different ML regression algorithms were evaluated, using all the samples from the two botanical origins generated during the adulteration process (D_48×4190_). Once again, the samples were divided into two subsets: (I) The training set, which contained 75% of the samples and was used to create the regression models. (II) The test set, which contained 25% of the remaining samples and was used as an external validation. Additionally, it was ensured that the partition was performed in a balanced way and at least one sample of each percentage was in the test set.

The regression ML models evaluated were PLS, shrinkage methods (lasso, ridge, and elastic net), SVR, and RF. Prior to developing these regression algorithms, a data preprocessing step was performed which involved selecting variables in the training set by applying the Boruta algorithm. In general, variable selection helps build more reliable and efficient regression models, leading to better insights and more accurate predictions. In addition, it is essential for improving model interpretability, enhancing performance, ensuring computational efficiency, and addressing multicollinearity. In particular, the Boruta algorithm is based on random forest models and deals with correlated features, nonlinear relationships, and noisy data. Moreover, in this scenario, the Boruta algorithm has been used due to its notable efficacy in handling high-dimensional datasets that encompass a large number of features. The number of wavelengths related to the response variable (percentage of adulteration) was considerably reduced from 4190 to 90 wavelengths. Specifically, this Boruta algorithm identified 90 significant variables for predicting adulteration, rejecting 4078 variables and considering 22 variables as tentative. Once the significant variables were selected, the aforementioned models were trained. Table 2 summarizes the main performance statistics obtained. It should be noted that, for optimizing the different hyperparameters of the models, leave-one-out cross-validation (LOOCV) was used. This optimization involves iteratively training the model on all but one sample and then testing it on the left-out sample. This process is repeated for each sample in the dataset, resulting in multiple iterations. LOOCV provides a reliable assessment of the model’s generalization ability as each sample is used as a validation set once. The final performance of the model is determined by the average result from all iterations. In this scenario, LOOCV proves to be particularly advantageous and preferable over 5-fold cross-validation due to the smaller size of the dataset. Additionally, LOOCV offers the advantage of individually testing each sample and percentage of adulteration, enhancing the thoroughness of the evaluation process. Again, this validation is used to avoid overfitting during hyperparameter optimization, and the test set is also used to check the error. In all validations, the root mean square error (RMSE) and the coefficient of determination (R^2^) were calculated.

#### 3.3.1. Partial Least Squares (PLS)

The optimal number of principal components (from 1 to 20) was evaluated through LOOCV in the training set. The best result was obtained for 8 components where the RMSE value was 3.961 and the R^2^ was 0.921. The model created with the 8 components achieved an RMSE of 2.102 and 2.784 as well as an R^2^ of 0.981 and 0.976 in the training and test sets, respectively.

#### 3.3.2. Shrinkage Methods

##### Lasso

In this linear regression, a penalty is applied, which reduces the regression coefficients to zero for irrelevant variables, leading to a simpler and easier to interpret model. However, there is a hyperparameter called lambda (*λ*) that controls the degree of penalty. For the optimization of this, LOOCV was again used on the training set by testing values in the sequence from −5 to 5 with intervals of 0.1. The best result was obtained with a λ value of 1 which led to an RMSE of 3.138 and an R^2^ of 0.964 in the LOOCV itself. However, the RMSE and R^2^ for the training and test sets were as follows: for the training set, the RMSE was 1.983 and the R^2^ was 0.996, while for the test set, the RMSE was 2.081 and the R^2^ was 0.986.

##### Ridge

In this linear regression, a penalty is applied again, but unlike lasso, the coefficients are not reduced to 0 and, therefore, are not excluded. In this way, the amount of noise in the data is minimized and the accuracy of the predictions is improved. The optimization of the lambda hyperparameter was carried out in the same way as in lasso, and the best results were obtained for a value of 4. This model resulted in an RMSE of 5.312 and an R^2^ of 0.871 in the LOOCV. The performance for the training and test sets was an RMSE of 5.071 and 12.352, and an R^2^ of 0.885 and 0.723, respectively. Although the RMSE and R^2^ indicated a high correlation between the percentage of adulteration and the variables, the performance was considerably inferior to that obtained using lasso and PLS. Therefore, it would be discarded for quantifying the level of adulteration in honey.

##### Elastic Net

This model combines ridge and lasso regression to balance variable selection and coefficient reduction. To achieve this, a new hyperparameter is introduced, requiring the adjustment of lambda, which controls the degree of penalty, and alpha (*α*), which controls the degree of influence of each penalty (ridge and lasso). The best combination of hyperparameters was obtained for a *λ* of 0.22 and an *α* of 0.53, with an RMSE of 3.501 and an R^2^ of 0.952 for LOOCV. With these values, the RMSE was 3.031 and 3.586 and the R^2^ was 0.969 and 0.939 for the training and test sets, respectively.

The obtained results were superior to ridge; however, among the three shrinkage methods, lasso achieved better results, making it the optimal model for quantifying the adulterant.

#### 3.3.3. Support Vector Regression (SVR) with Gaussian Kernel

Like the SVM algorithm for classification purposes, SVR requires tuning the hyperparameters *C* and *γ*, and, therefore, the same optimization method was followed. Additionally, a new hyperparameter controlling the learning rate, called epsilon (*ε*), was introduced and in this case, it was kept constant at 0.1. The optimal values for *C* and *γ* were 64 (log_2_*C* = 6) and 5.52 × 10^−3^ (log_2_*γ* = −7.5), respectively. The resulting model achieved an R^2^ of 0.994 and 0.991 for the training and test set, respectively, with an RMSE of 1.432 and 1.894. These demonstrated the potential of this model to predict the percentage of adulteration.

#### 3.3.4. Random Forest Regression (RF)

The number of trees was kept at 500 and the *mtry* value was optimized by testing values from 1 to 30. The model with the lowest RMSE presented a *mtry* of 5, resulting in an R^2^ of 0.851 and an RMSE of 7.328 for LOOCV. In the training and test set, an RMSE of 2.754 and 8.475 were obtained, as well as an R^2^ of 0.985 and 0.813, respectively. As can be seen, there was an increase in the RMSE in the test set, indicating that the model suffers from some overfitting.

For the overall quantification of the adulterant, the results were satisfactory, especially for the SVR, lasso, and PLS models. The obtained result indicated a slightly superior potential of the SVR model with an RMSE of 1.894 on the test set. Again, this emphasized the importance of analyzing a larger number of samples, as it would contribute to improving the performance metrics. By training the models with a broader range of spectral information, the variations attributed to sample preparation and Vis-NIRs analysis would be mitigated. It should be noted that there are studies based on spectroscopy data in which better results were obtained with PLS than with SVR [53], while in other cases, the opposite is reported [54] or even similar performances [55].

### 3.4. Application Development

In order to automatize the data analysis and share with other users, a web application has been developed. The app incorporates both SVM and SVR models, which have shown outstanding performance in detecting and quantifying the adulterant. The link to access the application is provided below:

https://agr291.shinyapps.io/App_Honey/ (accessed on 20 June 2023).

The operation of this application is quite simple, and the user would simply need to upload the Excel file generated directly from the sample analysis using Vis-NIRs. By clicking the “submit” button, predictions are made immediately. This methodology automates the data analysis process, allowing for obtaining the results within seconds, which is a great advantage. Considering the significant challenge of generalizing this methodology to encompass honeys of diverse botanical origins or those adulterated with various substances, the models uploaded to the website will be updated as more samples are analyzed. In this way, more information can be incorporated into the models, covering a greater variability. Nevertheless, it should be noted that the current models have been trained exclusively on samples of orange blossom and sunflower honeys, with lower-cost honey mixtures as adulterants. As a result, these models may not be capable of identifying other honeys of different botanical origins or detect alternative adulterants such as syrups or sugars.

## 4. Conclusions

The potential of Vis-NIRs in combination with ML tools has been demonstrated for detecting and quantifying in an automated way adulteration in high-quality honeys of different botanical origins (orange blossom and sunflower) when adulterated with other low-cost honeys. The exploratory analysis showed a clustering trend according to botanical origin, as well as the presence of adulteration. Regarding the supervised analysis, most of the classification models correctly detected 100% of the adulterations in the test set. In the case of quantification, the best results were obtained for the SVR model with an RMSE of 1.894 and an R^2^ of 0.991 for the test set. This allows the method to be able not only to detect adulterations in honey with other low-price honeys, but also to quantify them. Finally, an application that automates the detection of adulteration in honey has been developed. This web application facilitates other users to process the data even without ML knowledge, thus saving time and interpretation efforts. However, one of the main challenges of using this methodology to detect and quantify adulterations is the dynamic nature of the food industry, which constantly introduces new adulterants, and sample heterogeneity. As a result, the models must continually learn and be adapted. Consequently, the proposed website will be regularly updated as new samples are analyzed to align with the evolving requirements of the food industry.

## Figures and Tables

**Table 1:** Accuracy obtained by the classification models trained for the detection of adulterations using the complete data matrix (D_70×4190_).

Models	Hyperparameter	CV-5-Fold Accuracy	Training Set Accuracy	Test Set Accuracy
**SVM GAUSSIAN**	*C* = 1 *Y* = 1.38 × 10^−3^	100%	100%	100%
**SVM LINEAL**	*C* = 9.77 × 10^−4^	100%	100%	100%
**LDA**	-	-	100%	94.12%
**RF**	*mtry* = 64 ntree = 500	-	100%	100 %

**Table 2:** Results obtained on the main performance metrics of the regression models trained for the global quantification of adulterant.

MODELS	Hyperparameters	LOOCV Performance	Training Set Performance	Test Set Performance
**PLS**	8 principal components	RMSE = 3.961 R^2^ = 0.921	RMSE = 2.102 R^2^ = 0.981	RMSE = 2.784 R^2^ = 0.976
**SVR**	*C* = 64 *Y* = 5.52 × 10^−3^	RMSE = 2.075 R^2^ = 0.987	RMSE = 1.432 R^2^ = 0.994	RMSE = 1.894 R^2^ = 0.991
**RF**	*mtry* = 5	RMSE = 7.328 R^2^ = 0.851	RMSE = 2.754 R^2^ = 0.985	RMSE = 8.475 R^2^ = 0.813
**LASSO**	*λ* = 1	RMSE = 3.138 R^2^ =0.964	RMSE = 1.983 R^2^ = 0.996	RMSE = 2.081 R^2^ = 0.986
**RIDGE**	*λ* = 4	RMSE = 5.312 R^2^ = 0.871	RMSE = 5.071 R^2^ = 0.885	RMSE = 12.352 R^2^ = 0.723
**ELASTIC NET**	*λ* = 0.22 *α* = 0.53	RMSE = 3.501 R^2^ = 0.952	RMSE = 3.031 R^2^ = 0.969	RMSE = 3.586 R^2^ = 0.939

## Data Availability

The data presented in this study are contained within the article.

## Supplementary Materials

The following supporting information can be downloaded at: https://www.mdpi.com/article/10.3390/foods12132491/s1, Table S1: correlation between cophenetic distance and distance matrix for different HCA clustering methods using the NIR spectrum (with first derivative and Savitzky–Golay filter) of all honey samples (D_70×4190_).

## References

[1] Ilia G., Simulescu V., Merghes P., Varan N. (2021). The Health Benefits of Honey as an Energy Source with Antioxidant, Antibacterial and Antiseptic Effects. Sci. Sport..

[2] European Parliament Committee on the Environment, Public Health and Food Safety (2013). REPORT on the Food Crisis, Fraud in the Food Chain and the Control Thereof.

[3] European Parliament (2014). Directive 2014/63/EU of the European Parliament and of the Council Amending Council Directive 2001/110/EC Relating to Honey.

[4] Se K.W., Wahab R.A., Syed Yaacob S.N., Ghoshal S.K. (2019). Detection Techniques for Adulterants in Honey: Challenges and Recent Trends. J. Food Compos. Anal..

[5] Ilyasov R.A., Kosarev M.N., Neal A., Yumaguzhin F.G. (2015). Burzyan Wild-Hive Honeybee A.M. Mellifera in South Ural. Bee World.

[6] Soares S., Amaral J.S., Beatriz M., Oliveira P.P., Mafra I. (2017). A Comprehensive Review on the Main Honey Authentication Issues: Production and Origin. Compr. Rev. Food Sci. Food Saf..

[7] Zhang G., Abdulla W. (2022). On Honey Authentication and Adulterant Detection Techniques. Food Control.

[8] Cotte J.F., Casabianca H., Giroud B., Albert M., Lheritier J., Grenier-Loustalot M.F. (2004). Characterization of Honey Amino Acid Profiles Using High-Pressure Liquid Chromatography to Control Authenticity. Anal. Bioanal. Chem..

[9] García-Seval V., Martínez-Alfaro C., Saurina J., Núñez O., Sentellas S. (2022). Characterization, Classification and Authentication of Spanish Blossom and Honeydew Honeys by Non-Targeted HPLC-UV and Off-Line SPE HPLC-UV Polyphenolic Fingerprinting Strategies. Foods.

[10] Wang S., Guo Q., Wang L., Lin L., Shi H., Cao H., Cao B. (2015). Detection of Honey Adulteration with Starch Syrup by High Performance Liquid Chromatography. Food Chem..

[11] Xue X., Wang Q., Li Y., Wu L., Chen L., Zhao J., Liu F. (2013). 2-Acetylfuran-3-Glucopyranoside as a Novel Marker for the Detection of Honey Adulterated with Rice Syrup. J. Agric. Food Chem..

[12] Yilmaz M.T., Tatlisu N.B., Toker O.S., Karaman S., Dertli E., Sagdic O., Arici M. (2014). Steady, Dynamic and Creep Rheological Analysis as a Novel Approach to Detect Honey Adulteration by Fructose and Saccharose Syrups: Correlations with HPLC-RID Results. Food Res. Int..

[13] Sotiropoulou N.S., Xagoraris M., Revelou P.K., Kaparakou E., Kanakis C., Pappas C., Tarantilis P. (2021). The Use of SPME-GC-MS IR and Raman Techniques for Botanical and Geographical Authentication and Detection of Adulteration of Honey. Foods.

[14] Wei Q., Sun J., Guo J., Li X., Zhang X., Xiao F. (2023). Authentication of Chaste Honey Adulterated with High Fructose Corn Syrup by HS-SPME-GC-MS Coupled with Chemometrics. LWT.

[15] Kružík V., Grégrová A., Rajchl A., Čížková H. (2017). Study on Honey Quality Evaluation and Detection of Adulteration by Analysis of Volatile Compounds. J. Apic. Sci..

[16] Aliaño-González M.J., Ferreiro-González M., Espada-Bellido E., Barbero G.F., Palma M. (2020). Novel Method Based on Ion Mobility Spectroscopy for the Quantification of Adulterants in Honeys. Food Control.

[17] Arroyo-Manzanares N., García-Nicolás M., Castell A., Campillo N., Viñas P., López-García I., Hernández-Córdoba M. (2019). Untargeted Headspace Gas Chromatography—Ion Mobility Spectrometry Analysis for Detection of Adulterated Honey. Talanta.

[18] Aliaño-González M.J., Ferreiro-González M., Espada-Bellido E., Palma M., Barbero G.F. (2019). A Screening Method Based on Headspace-Ion Mobility Spectrometry to Identify Adulterated Honey. Sensors.

[19] Cagliani L.R., Maestri G., Consonni R. (2022). Detection and Evaluation of Saccharide Adulteration in Italian Honey by NMR Spectroscopy. Food Control.

[20] Rachineni K., Rao Kakita V.M., Awasthi N.P., Shirke V.S., Hosur R.V., Chandra Shukla S. (2022). Identifying Type of Sugar Adulterants in Honey: Combined Application of NMR Spectroscopy and Supervised Machine Learning Classification. Curr. Res. Food Sci..

[21] Mohamadzade Namin S., Yeasmin F., Choi H.W., Jung C. (2022). DNA-Based Method for Traceability and Authentication of *Apis cerana* and *A. Dorsata* Honey (Hymenoptera: Apidae), Using the NADH Dehydrogenase 2 Gene. Foods.

[22] Truong A., Kim S., Yoon B. (2022). Determination of Honey Adulterated with Corn Syrup by Quantitative Amplification of Maize Residual DNA Using Ultra-rapid Real-time PCR. J. Sci. Food Agric..

[23] Machuca G., Staforelli J., Rondanelli-Reyes M., Garces R., Contreras-Trigo B., Tapia J., Sanhueza I., Jara A., Lamas I., Troncoso J.M. (2022). Hyperspectral Microscopy Technology to Detect Syrups Adulteration of Endemic Guindo Santo and Quillay Honey Using Machine-Learning Tools. Foods.

[24] Pirmoradi M., Mostafaei M., Naderloo L., Javadikia H. (2022). Modeling Honey Adulteration by Processing Microscopic Images Using Artificial Intelligence Methods. J. Agric. Sci. Technol..

[25] Devi A., Jangir J., Appaiah K.A. (2018). Chemical Characterization Complemented with Chemometrics for the Botanical Origin Identification of Unifloral and Multifloral Honeys from India. Food Res. Int..

[26] Berghian-Grosan C., Hategan A.R., David M., Magdas D.A. (2023). Untargeted Metabolomic Analysis of Honey Mixtures: Discrimination Opportunities Based on ATR-FTIR Data and Machine Learning Algorithms. Microchem. J..

[27] Rios-Corripio M.A., Rojas-López M., Delgado-Macuil R. (2012). Analysis of Adulteration in Honey with Standard Sugar Solutions and Syrups Using Attenuated Total Reflectance-Fourier Transform Infrared Spectroscopy and Multivariate Methods. CyTA—J. Food.

[28] Riswahyuli Y., Rohman A., Setyabudi F.M.C.S., Raharjo S. (2020). Indonesian Wild Honey Authenticity Analysis Using Attenuated Total Reflectance-Fourier Transform Infrared (ATR-FTIR) Spectroscopy Combined with Multivariate Statistical Techniques. Heliyon.

[29] Li Q., Zeng J., Lin L., Zhang J., Zhu J., Yao L., Wang S., Yao Z., Wu Z. (2020). Low Risk of Category Misdiagnosis of Rice Syrup Adulteration in Three Botanical Origin Honey by ATR-FTIR and General Model. Food Chem..

[30] Li Y., Yang H. (2012). Honey Discrimination Using Visible and Near-Infrared Spectroscopy. ISRN Spectrosc..

[31] Gok S., Severcan M., Goormaghtigh E., Kandemir I., Severcan F. (2015). Differentiation of Anatolian Honey Samples from Different Botanical Origins by ATR-FTIR Spectroscopy Using Multivariate Analysis. Food Chem..

[32] Chen L., Wang J., Ye Z., Zhao J., Xue X., Vander Heyden Y., Sun Q. (2012). Classification of Chinese Honeys According to Their Floral Origin by near Infrared Spectroscopy. Food Chem..

[33] Valinger D., Longin L., Grbeš F., Benković M., Jurina T., Gajdoš Kljusurić J., Jurinjak Tušek A. (2021). Detection of Honey Adulteration—The Potential of UV-VIS and NIR Spectroscopy Coupled with Multivariate Analysis. LWT.

[34] Yang X., Guang P., Xu G., Zhu S., Chen Z., Huang F. (2020). Manuka Honey Adulteration Detection Based on Near-Infrared Spectroscopy Combined with Aquaphotomics. LWT.

[35] Huang F., Song H., Guo L., Guang P., Yang X., Li L., Zhao H., Yang M. (2020). Detection of Adulteration in Chinese Honey Using NIR and ATR-FTIR Spectral Data Fusion. Spectrochim. Acta A Mol. Biomol. Spectrosc..

[36] Aliaño-González M.J., Ferreiro-González M., Espada-Bellido E., Palma M., Barbero G.F. (2019). A Screening Method Based on Visible-NIR Spectroscopy for the Identification and Quantification of Different Adulterants in High-Quality Honey. Talanta.

[37] Ferreiro-González M., Espada-Bellido E., Guillén-Cueto L., Palma M., Barroso C.G., Barbero G.F. (2018). Rapid Quantification of Honey Adulteration by Visible-near Infrared Spectroscopy Combined with Chemometrics. Talanta.

[38] Li S., Zhang X., Shan Y., Su D., Ma Q., Wen R., Li J. (2017). Qualitative and Quantitative Detection of Honey Adulterated with High-Fructose Corn Syrup and Maltose Syrup by Using near-Infrared Spectroscopy. Food Chem..

[39] Bázár G., Romvári R., Szabó A., Somogyi T., Éles V., Tsenkova R. (2016). NIR Detection of Honey Adulteration Reveals Differences in Water Spectral Pattern. Food Chem..

[40] Kumaravelu C., Gopal A. (2015). Detection and Quantification of Adulteration in Honey through Near Infrared Spectroscopy. Int. J. Food Prop..

[41] Mishra P., Biancolillo A., Roger J.M., Marini F., Rutledge D.N. (2020). New Data Preprocessing Trends Based on Ensemble of Multiple Preprocessing Techniques. TrAC Trends Anal. Chem..

[42] Calle J.L.P., Barea-Sepúlveda M., Ruiz-Rodríguez A., Álvarez J.Á., Ferreiro-González M., Palma M. (2022). Rapid Detection and Quantification of Adulterants in Fruit Juices Using Machine Learning Tools and Spectroscopy Data. Sensors.

[43] Wickham H. (2016). Ggplot2: Elegant Graphics for Data Analysis.

[44] Stevens A., Ramirez-Lopez L. (2020). An Introduction to the Prospectr Package.

[45] Kassambara A., Mundt F. (2020). factoextra: Extract and Visualize the Results of Multivariate Data Analyses. R Package Version 1.0.7. https://CRAN.R-project.org/package=factoextra.

[46] Kuhn M. (2008). Building Predictive Models in R Using the caret Package. J. Stat. Softw..

[47] Chang W., Cheng J., Allaire J., Sievert C., Schloerke B., Xie Y., Allen J., McPherson J., Dipert A., Borges B. (2022). _shiny: Web Application Framework for R. R Package Version 1.7.4. https://CRAN.R-project.org/package=shiny.

[48] Calle J.L.P., Ferreiro-González M., Ruiz-Rodríguez A., Fernández D., Palma M. (2022). Detection of Adulterations in Fruit Juices Using Machine Learning Methods over FT-IR Spectroscopic Data. Agronomy.

[49] Calle J.L.P., Falatová B., Aliaño-González M.J., Ferreiro-González M., Palma M. (2022). Machine Learning Approaches over Ion Mobility Spectra for the Discrimination of Ignitable Liquids Residues from Interfering Substrates. Talanta Open.

[50] Zhu X., Li S., Shan Y., Zhang Z., Li G., Su D., Liu F. (2010). Detection of Adulterants Such as Sweeteners Materials in Honey Using Near-Infrared Spectroscopy and Chemometrics. J. Food Eng..

[51] Heidari A., Navimipour N.J., Jamali M.A.J., Akbarpour S. (2023). A Green, Secure, and Deep Intelligent Method for Dynamic IoT-Edge-Cloud Offloading Scenarios. Sustain. Comput. Inform. Syst..

[52] Heidari A., Javaheri D., Toumaj S., Navimipour N.J., Rezaei M., Unal M. (2023). A New Lung Cancer Detection Method Based on the Chest CT Images Using Federated Learning and Blockchain Systems. Artif. Intell. Med..

[53] Elhamdaoui O., El Orche A., Cheikh A., Mojemmi B., Nejjari R., Bouatia M. (2020). Development of Fast Analytical Method for the Detection and Quantification of Honey Adulteration Using Vibrational Spectroscopy and Chemometrics Tools. J. Anal. Methods Chem..

[54] Šnurkovič P. (2013). Quality Assessment of Fruit Juices by NIR Spectroscopy. Acta Univ. Agric. Silvic. Mendel. Brun..

[55] Li C., He M., Cai Z., Qi H., Zhang J., Zhang C. (2023). Hyperspectral Imaging with Machine Learning Approaches for Assessing Soluble Solids Content of Tribute Citru. Foods.

