# Supplementary material for: Rapid and Automated Method for Detecting and Quantifying Adulterations in High-Quality Honey Using Vis-NIRs in Combination with Machine Learning
Source: Foods. 2023 Jun 26;12(13):2491. doi: 10.3390/foods12132491 (PMC10340210; doi:10.3390/foods12132491)
Supplement: Supplementary file 1 [file foods-12-02491-s001.zip › foods-2451610-supplementary.pdf]

**Table S1**: Correlation between Cophenetic distance and distance matrix for different HCA clustering methods using the NIR spectrum (with first derivative and Savitzky-Golay filter) of all honey samples ($D_{70x4190}$).

| Linkage method | Cophenetic distance |
|---|---|
| **Single** | 0.8721 |
| **Complete** | 0.8853 |
| **Average** | 0.8279 |
| **Ward** | 0.9053 |
| **Centroid** | 0.8778 |
